# Mitomycin C, vinblastine and cisplatin (MVP): an active and well-tolerated salvage regimen for advanced breast cancer

**DOI:** 10.1038/sj.bjc.6602367

**Published:** 2005-02-01

**Authors:** A Urruticoechea, C D Archer, L A Assersohn, R K Gregory, M Verrill, R Mendes, G Walsh, I E Smith, S R D Johnston

**Affiliations:** 1 Department of Medicine – Breast Unit, Royal Marsden NHS Trust, London and Surrey, England

**Keywords:** anthracycline, metastatic breast cancer, chemotherapy, cisplatin

## Abstract

This phase II study assessed the clinical efficacy and tolerability of a combination of mitomycin C, vinblastine and cisplatin in patients with metastatic breast cancer (MBC) previously treated with chemotherapy. A total of 87 patients with MBC, most of whom had been exposed to anthracyclines (92%) and/or taxanes (29%) in the adjuvant and/or metastatic setting, were treated with mitomycin C (8 mg m^−2^ day 1 cycles 1, 2, 4 and 6), vinblastine (6 mg m^−2^ day 1) and cisplatin (50 mg m^−2^ day 1) repeated each 21 days for a maximum of six cycles. The overall response rate (ORR) was 32% (95% CI: 22–42%) with 31% partial response (PR) and one complete response (CR). Stable disease (SD) rate was 21% (95% CI: 12–29%). There was no statistically significant difference in the ORR when MVP was given as the first-line treatment for MBC *vs* second or subsequent line (38 *vs* 30%, *P*=0.6), or between patients with an early (<6 months) *vs* late (>6 months) relapse post-anthracyclines (30 *vs* 52%, *P*=0.3). Toxicity profile was mild. This platinum-based chemotherapy is an effective, well-tolerated and low-cost regimen for patients with MBC, including those pretreated with anthracyclines.

Breast cancer is the most frequent malignant disease among Western women. Despite treatment following early stage diagnosis, a high percentage of patients develop metastatic disease.

Most patients now receive anthracycline-based chemotherapy regimens in the adjuvant setting, and an increasing number of them receive taxanes either in this setting or as first-line therapy for metastatic disease. The majority of patients developing or presenting with metastatic disease show a good performance status, making them eligible for systemic cytotoxic drug treatments. The development of new chemotherapy regimens is thus needed to treat anthracycline and/or taxane-pretreated patients.

A number of previous studies (Kolaric and Roth, 1983; Sledge *et al*, 1988; Smith and Talbot, 1992) have demonstrated the efficacy of cisplatin-based chemotherapy regimens both in previously untreated patients with metasatic breast cancer, and following anthracycline failure (Vassilomanolakis *et al*, 2000; Mustacchi *et al*, 2002). However, the widespread use of platinum-based treatments has been undermined by the alleged lower tolerability of these regimens and the requirement for prolonged infusion protocols.

Some recent preclinical data have suggested a potential higher benefit for younger patients with BRCA-1 mutation from platinum-based chemotherapy, given the drug's mechanism of action as a DNA-damaging agent to which these patients’ tumours may be especially sensitive (Husain *et al*, 1998; Bhattacharyya *et al*, 2000; Tassone *et al*, 2003). Some studies have also shown synergy between cisplatinum and trastuzumab in HER-2-positive breast cancer (Pegram *et al*, 1998; Konecny *et al*, 1999). Hence, there is a rationale to develop an effective and well-tolerated platinum-based regimen for metastatic breast cancer (MBC).

Our previous experience with a combination of cisplatin, vinblastine and mitomycin C (MVP) in lung cancer patients (Ellis *et al*, 1995) led us to run a prospective phase II trial to study this regimen in women with MBC, including those pretreated with anthracyclines. In addition, a group of patients previously treated with taxanes were also included.

## PATIENTS AND METHODS

### Eligibility

Patients were required to have received prior chemotherapy either in the adjuvant setting or as first-line treatment with a minimal period of 4 weeks free of disease progression. All patients had either measurable or evaluable disease. Nonmeasurable but evaluable disease was defined as malignant disease evident on physical or radiological examination, but not measurable by ruler or callipers, for example, locally advanced chest wall disease evaluated by photography, or multiple small (<1 cm) skin nodules. Patients were required to have Eastern Cooperative Oncology Group performance status ⩽2, adequate bone marrow reserve (WBC count ⩾3.5 × 10^9^ l^−1^, platelets ⩾100 × 10^12^ l^−1^ and haemoglobin ⩾10 g dl^−1^) and satisfactory renal function (creatinine clearance ⩾60 ml min^−1^ measured by ^51^Cr EDTA or Cockcroft and Gault estimation). Liver function tests were allowed up to twice normal values, or five times if liver metastases were present. No prior cisplatin chemotherapy was allowed, but there was no limit placed on the number of previous chemotherapy regimens or endocrine treatments received. The study was approved by the Royal Marsden Hospital Ethics Committee and all patients gave written informed consent.

### Treatment schedule

The treatment regimen was similar to that previously reported for patients with non-small-cell lung cancer (11): mitomycin C 8 mg m^−2^ i.v. day 1 (cycles 1, 2, 4 and 6 only), vinblastine 6 mg m^−2^ (max 10 mg) i.v. day 1 and cisplatin 50 mg m^−2^ day 1 of each cycle.

The hydration and diuretic treatment was as follows: *prior to cisplatin*: furosemide 40 mg i.v. and 1 l 0.9% NaCl with 20 mmol KCl and 10 mmol MgSO_4_ over 1 h; cisplatin dose was administered in 1 l 0.9% NaCl with 20 mmol KCl, 10 mmol MgSO_4_ over 4 h and manitol 100 ml 20% i.v; *post- cisplatin*: 1 l 0.9% NaCl with 20 mmol KCl and 10 mmol MgSO_4_ over 2 h and then 500 ml 0.9% NaCl with 10 mmol KCl and 5 mmol MgSO_4_ over 1 h. Antiemetic treatment was delivered prechemotherapy with domperidone 20 mg, granisetron 1 mg and dexamethasone 8 mg i.v. and postchemotherapy with domperidone 10–20 mg p.o. qds for 3 days and dexamethasone 4 mg p.o. tds for 3 days.

The schedule was given as either an in-patient (overnight admission), or as a day-case and repeated every 3 weeks for a maximum of six cycles.

### Tumour response assessment

Toxicity recording and clinical assessment of tumour response as well as clinical examination and full blood count test was performed prior to each cycle. Formal radiological assessment of response by computed tomography or X-ray was undertaken prior to study entry, after three cycles and after six cycles or at the end of treatment if earlier. Standard WHO criteria were used to define response (this study predated the routine use of RECIST criteria).

### Treatment modifications

Treatment was delayed for 1 week if the white cell count was<3.0 × 10^9^ l^−1^, or platelets <100 × 10^12^ l^−1^. Renal function was assessed at each cycle with plasma urea and creatinine, and Cockcroft and Gault estimation. If creatinine clearance fell between 40 and 60 ml min^−1^, then the cisplatin dose was equivalent to the creatinine clearance and, if creatinine clearance fell to <40 ml min^−1^, then cisplatin was replaced by carboplatin at a dose of AUC × 5 (AUC=creatinine clearance or EDTA+25) (Smith *et al*, 2001).

## RESULTS

### Patient characteristics

Between September 1996 and May 2002, 87 patients diagnosed with MBC were treated with MVP in this phase II study. The characteristics of patients are summarised in Table 1. A total of 80 (92%) had previously been treated with anthracyclines, and 25 (29%) with taxanes. Total of 60 patients (69%) had received prior adjuvant chemotherapy, and 63 (72%) had already received cytotoxic treatment for MBC. All patients had received at least one prior chemotherapy regimen.

### Clinical efficacy

Data on response to MVP combination are shown in Table 2 analysed on an intention-to-treat (ITT) basis. In all, 27 patients (31%) showed a partial response (PR), and one (1%) a complete response (CR) resulting in an overall response rate (ORR) of 32% (95% CI: 22–42%). A total of 18 (21%, 95% CI: 12–29%) patients achieved stable disease (SD) as the best response; median duration 20 weeks (95% CI: 12–58 weeks). When MVP was given as the first-line treatment for MBC, an objective response was obtained in nine out of 24 patients (38%, 95% CI: 18–57%)) compared with 19 out of 63 (30% (95% CI: 19–41%)) when given as second to sixth line (Table 2). No statistical difference was seen comparing these two groups (*P*=0.6).

In 31 patients, MVP was given on progression after an anthracycline-based treatment. These patients relapsed/progressed early (<6 months since completion of anthracycline therapy either in the metastatic or adjuvant setting), or later (>6 months). A total of 10 patients were in the former group and 21 in the latter. No difference in ORR was seen between early and late relapse groups (30 *vs* 52%, *P*=0.3). In the group of taxane pretreated patients (all had received taxane as first-line therapy for metasatic disease), 21 out of 25 were evaluable for response. In these patients, an ORR of 20% was achieved (95% CI: 4–36%), which does not reach statistical difference (*P*=0.06) when compared with the general group.

**Figure 1:**
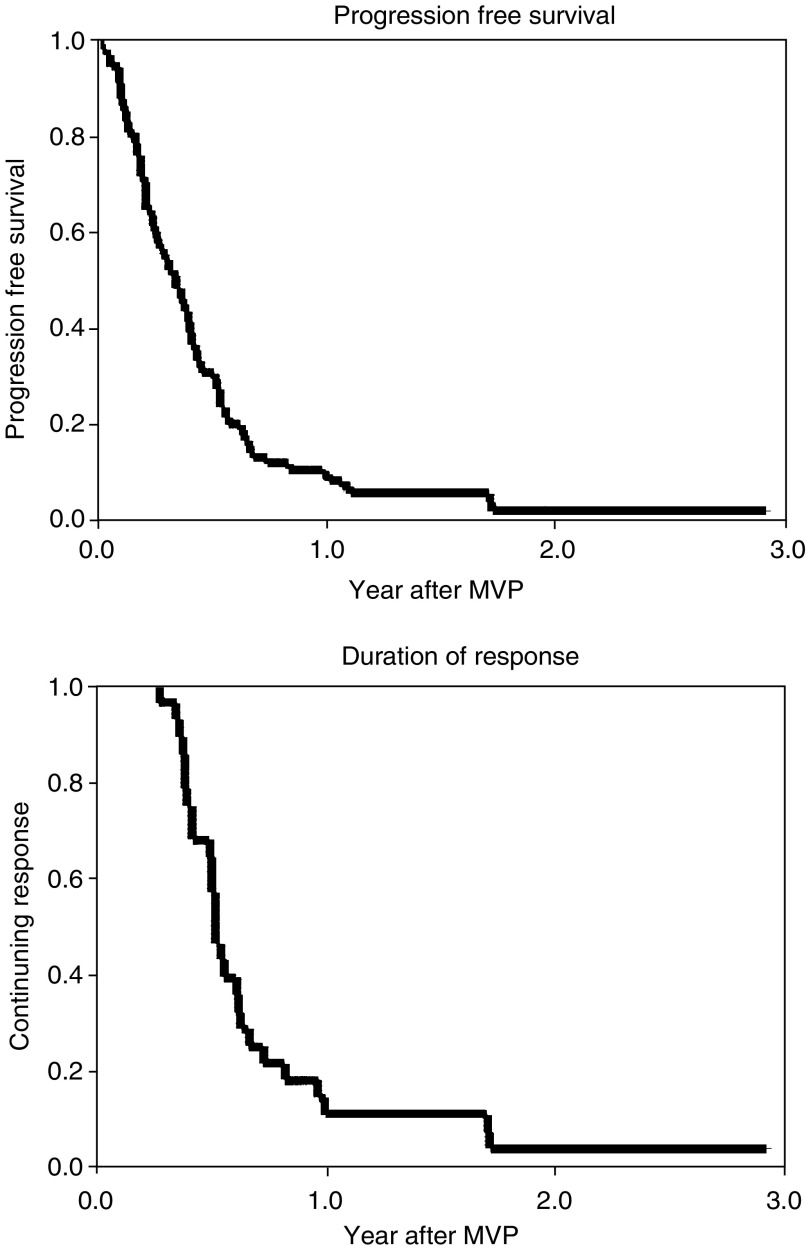
Progression-free survival and duration of response curves.

Overall, the median duration of objective response was 7 months (range: 4–23) in 27 out of the 28 responders; one patient remains in remission at 35+ months. The median progression-free survival was 4 months with a median overall survival of 8 months (Figure 1).

### Treatment and toxicity

In total, 350 cycles were delivered in 87 patients with a median number of 4.02 cycles per patient. Treatment was delivered either as an in-patient regimen with an overnight stay (the majority), or as a day-case treatment.

Data on toxicity are presented in Table 3. In all, 19 cycles out of a total of 350 were associated with febrile neutropenia (5.5% of cycles) in 16 patients (18% of patients). Other haematological toxicity was infrequent. The most common nonhaematological toxicity was lethargy with a 26% incidence of grade 3. Nausea and vomiting were not severe in most of the patients, with 11% presenting with grade 3 and none with grade 4 toxicity. Neuropathy was always mild and reversible with one only patient presenting with grade 3 toxicity.

A total of 41 patients required treatment delay at some point, mainly due to haematological toxicity. In all, 12 patients required a dose reduction of one or more drugs and 17 patients stopped treatment due to toxicity, five because of haematological toxicity and 12 because of nonhaematological toxicity (three cases of increased liver function tests, two of emesis, two of constipation, one of decreased glomerular filtration rate, one of acute ischaemic limb, one of lethargy, one of anxiety and one nonspecified). Nine patients received less than two cycles due to toxicity (haematological toxicity in three cases, constipation in two, emesis in one, raised liver function test in one and lethargy in two). No toxic deaths occurred.

### Drug cost

The actual drug cost for MVP calculated for a patient with 1.7 m^2^ body surface is around £80 (112€) per cycle.

## DISCUSSION

Chemotherapy remains a first-line treatment option for many patients diagnosed with metastatic breast cancer. With the increasing use of the most effective drugs in the adjuvant setting, there is an increasing need to evaluate novel regimens for MBC. The ideal treatment regimen should cause good symptom control through maximum tumour regression with a prolonged progression-free interval and minimal toxicity. Cost-effective regimens are also advantageous.

Anthracyclines (doxorubicin or epirubicin), either as single agent or in combination, are one of the most effective agents in the treatment of breast cancer and are increasingly used in the adjuvant setting in view of the survival benefit seen over non-anthracycline regimens (EBCTCG, 1998). Taxanes (paclitaxel and docetaxel) are the only drugs to have consistently shown similar or greater activity than doxorubicin in MBC (Chan *et al*, 1999), and have been established as the first option after failure of anthracyclines (Nabholtz *et al*, 1999). Recent studies (Buzdar *et al*, 2002; Nabholtz *et al*, 2002; Henderson *et al*, 2003) are leading to the progressive inclusion of taxanes in the adjuvant treatment schedules. This means that there will be an increasing number of MBC patients who present with good performance status, but are not candidates for retreatment with anthracyclines or taxanes because of either cardiac tolerance limits, or the likelihood of resistance to these therapies if they have relapsed within a 6-month period. Hence, the necessity for effective and well-tolerated palliative regimens.

Cisplatin has been combined with other agents in an attempt to derive an active regimen with no overlapping toxicities. Three studies have tested the combination of this drug with epirubicin plus or minus lonidamine and failed to show any substantial benefit from the addition of cisplatin (Gebbia *et al*, 1997; Dogliotti *et al*, 1998; Berruti *et al*, 2002). Despite being active (response rate of 73–82% in first line), the addition of cisplatin did not result in clinical benefit and the toxicity was higher. This latter finding was confirmed in a Danish study (Nielsen *et al*, 2000), although these authors found that survival in the epirubicin plus cisplatin arm was significantly longer than in the epirubicin only one.

When combined with taxanes, cisplatin has shown a response rate higher than 80% (Klaassen *et al*, 1998) although the additive toxicities, in particular neurotoxicity, have raised serious concerns about its feasibility (Wasserheit *et al*, 1996). Of note, most of the above-mentioned studies reporting high toxicity associated with cisplatin were based on cisplatin doses of 100 mg m^−2^ per cycle, while equally effective in response rates but better tolerance was seen when dose was between 50 and 60 mg m^−2^. Carboplatin has been tested in the same first-line setting and shown better tolerability in some studies, although less convincing results in terms of response rate (Kolaric and Vukas, 1991; Brambilla *et al*, 1993). Two recent reports showed that cisplatin in combination with vinka-alkaloids may be a very well-tolerated regimen as an alternative to taxanes after anthracyclines failure (Vassilomanolakis *et al*, 2000; Mustacchi *et al*, 2002).

Our series was commenced following evidence of good tolerance to MVP in lung cancer patients (Ellis *et al*, 1995), and shows very similar results to those reported above (Vassilomanolakis *et al*, 2000; Mustacchi *et al*, 2002). An ORR of 32% in a population of frequently heavily pretreated patients (92% of patients previously treated with anthracyclines and 28% with taxanes) is an encouraging result. The toxicity was mostly mild and tolerable with only 10% of patients stopping treatment due to toxicity prior to the third cycle. In addition, this is an active regimen that is considerably cheaper than taxane-based chemotherapy in this anthracycline-pretreated population with a drug cost per treatment cycle of £80, which compares favourably to approximate £1000 for taxanes-based chemotherapy. Nevertheless, the cost of overnight or day-case admission, and the specific antiemetic treatments, should all be considered in a more accurate comparative cost analyses.

In summary, MVP is a well-tolerated regimen with promising activity for advanced breast cancer, even in heavily pretreated patients. For certain groups of patients, its low toxicity makes it an alternative to taxanes, and MVP may also be a consideration after taxane failure. Prospective comparisons against other commonly used treatments in this setting such as vinorelbine or capecitabine are warranted.

## Figures and Tables

**Table 1:** Patients characteristics

Patients number		87
Age median in years (range)		50 (32–73)
*ER status*		
Positive		20
Negative		27
Unknown		40
		
*Previous adjuvant therapy*		
Chemo. only		15 (17%)
Chemo.+Tam		45 (52%)
Tamoxifen only		12 (14%)
None		15 (17%)
		
*No. of previous chemotherapy regimens for MBC*		
None		24 (28%)
1		36 (41%)
2		21 (24%)
3 or more		6 (7%)
		
Prior anthracyclines		80 (92%)
Prior taxanes		25 (29%)
		
*Site of metastasis*		
Soft tissue		80 (92%)
Lung		41 (47%)
Liver		37 (43%)
Pleura		33 (38%)
Bone		31 (36%)
CNS		2 (2%)

ER=oestrogen receptor; CNS=central nervous system; MBC=metastatic breast cancer; soft tissue includes breast and lymph nodes.

**Table 2:** Response data: (A) General group; (B) analyses by previous chemotherapy

**Response**	**No. of patients**	**% of all patients**	**% of evaluable patients**
*(A)*			
CR	1	1	1
PR	27	31	35
OR	28	32 (22–42%)	36 (26–47%)
SD	18	21	23
PD	31	36	40
NE	10	11	—
			
*(B)*			

**No previous chemo. for MBC**	**No. of patients**	**No. of responses**	**% of all patients**
Nil	24	9	38 (18–57)
1–5	63	19	30 (19–41)
Previous taxanes	25	5	20 (4–36%)

In brackets, 95% CI; CR=complete response; PR=partial response; SD=stable disease; PD=progressive disease; OR=overall response; NE=nonevaluable; chemo=chemotherapy; MBC=metastatic breast cancer.

**Table 3:** Worst toxicity, expressed as number of patients, ever observed in all 87 patients (WHO criteria)

**WHO grade**	**1+2 (%)**	**3 (%)**	**4 (%)**
Lethargy	50 (57)	22 (25)	0
Nausea/vomiting	48 (55)	11 (13)	0
Alopecia	34 (39)	8 (9)	0
Neuropathy	17 (20)	1 (1)	0
Stomatitis	34 (39)	2 (2)	0
Diarrhoea	16 (18)	5 (6)	0
Infection	22 (25)	22 (25)	0
Anaemia	50 (57)	7 (8)	1 (1)
Leucopenia	41 (47)	11 (13)	7 (8)
Neutropenia	20 (23)	10 (11)	18 (21)
Thrombocytopenia	15 (17)	9 (10)	3 (3)
